# Could a Two-Staged Repair Be the Solution to the Dilemma of Repair Timing for Severe Congenital Diaphragmatic Hernia Requiring Extracorporeal Membrane Oxygenation?

**DOI:** 10.3390/children11101255

**Published:** 2024-10-17

**Authors:** Akiko Yokoi

**Affiliations:** Department of Pediatric Surgery, Kobe Children’s Hospital, Kobe 650-0047, Japan; yokoi_kch@hp.pref.hyogo.jp; Tel.: +81-78-945-7300

**Keywords:** congenital diaphragmatic hernia (CDH), extracorporeal membranous oxygenation (ECMO), timing of repair

## Abstract

Purpose of review: Congenital diaphragmatic hernia (CDH) remains a significant challenge, particularly in severe cases with persistent pulmonary hypertension (PPHN) and hypoplastic lungs and heart. For patients unresponsive to conventional therapies, ECMO is required. While the surgical repair is relatively simple, determining the optimal timing for surgery in patients requiring ECMO is particularly challenging. This review explores the dilemma of surgical timing and proposes a two-staged approach: a reduction in herniated organs and the creation of a silo to relieve abdominal pressure before initiating ECMO, with defect closure following ECMO decannulation. Recent Findings: Studies support pre-, on-, and post-ECMO repair, each with its own risks and benefits. Pre-ECMO repair may enhance ECMO efficacy by relieving organ compression but poses risks due to instability. Post-ECMO repair is safer but may result in losing the chance to repair. On-ECMO repair has significant hemorrhage risks, but early repair with careful anticoagulation management is currently recommended. Recently, the author reported a successful case using a two-staged approach—reducing herniated organs and creating a silo before ECMO, followed by defect closure after ECMO decannulation—which suggests a potential alternative strategy for managing severe CDH. Summary: A two-staged approach may offer a solution for severe CDH patients requiring ECMO.

## 1. Introduction

Congenital diaphragmatic hernia (CDH) is one of the most challenging conditions for neonatologists and pediatric surgeons. The prevalence of CDH is 2.3 per 10,000 births [1]. CDH has a wide range of severities. Among 1975 CHD patients in the CDHSG (Congenital Diaphragmatic Hernia Study Group) registry 2007–2011, overall survival was 72% [1]. Recent advancements in the treatment of CDH, including techniques such as gentle ventilator permissive hypercapnia, high-frequency oscillatory ventilation (HFOV), inhaled nitric oxide (iNO), Extracorporeal Membrane Oxygenation (ECMO), pulmonary vasodilators, and fetal intervention, have resulted in a slight decrease in mortality. However, more than a quarter of patients still do not survive [2].

The pathology of CDH involves pulmonary hypoplasia and persistent pulmonary hypertension of the newborn (PPHN). Early postnatal cardiac function is also associated with mortality and ECMO use in CDH. Cardiac function ultimately affects pulmonary circulation, and low cardiac output raises pulmonary arterial resistance, leading to the deterioration of PPHN [3]. Although pulmonary hypoplasia is primarily due to a developmental abnormality of the pleuroperitoneal canal during diaphragm development, as shown in the Nitrofen animal model, the compression caused by herniated abdominal viscera is believed to interfere further with lung development after birth until CDH is repaired [4]. Most CDH cases involve mild-to-moderate left heart hypoplasia prenatally, which improves after birth and repair. However, in severe cases of left heart hypoplasia, the risk of mortality increases [5].

The surgical procedure for repairing CDH is relatively simple: a reduction in intrathoracic herniated organs into the abdomen and closure of the defect with or without the use of a prosthetic patch and/or muscle interposition. However, determining the optimal timing of surgery is profoundly challenging, especially for severe CDH cases requiring ECMO. Surgery relieves compression on the lung and heart by removing herniated intrathoracic organs, thereby improving lung compliance and cardiac function [6]. However, in severe cases, surgery may worsen PPHN. Vasoconstriction after surgery may exacerbate pulmonary hypertension [7]. The deforming thoracic cavity and increasing abdominal pressure contribute to postoperative deterioration [8]. Thus, determining the optimal timing of surgery is incredibly challenging, and studies have shown that timing has not contributed significantly to the outcome in high-risk CDH cases [9].

Moreover, although ECMO is the last resort for severe CDH patients who fail conventional therapy, it has not been proven to provide a survival advantage in managing CDH [10,11], even though it has been shown to be beneficial in improving the survival of neonates with other forms of respiratory failure [12]. One reason may be that repair during ECMO carries a higher risk of surgical complications compared to non-ECMO repair. Specifically, anticoagulant therapy during ECMO increases the risk of hemorrhage, which can be fatal for newborns. Reports indicate that the risk of hemorrhage during ECMO is significantly higher than during non-ECMO repair (29% vs. 2%) [13].

Recently, the author reported a two-staged approach for severe CDH requiring ECMO [14]. Briefly, the patient underwent laparotomy for the reduction in intrathoracic herniated abdominal organs, and the defect was left open, with a silo created to alleviate abdominal pressure right before ECMO cannulation. PPHN was successfully improved during ECMO, and after decannulation, the defect was closed with an abdominal muscle flap. The patient had a good outcome without neuroglial sequelae.

This review will address the current dilemma regarding the timing of repair for patients with severe CDH requiring ECMO and propose the potential benefits of a two-staged approach for this challenging condition.

## 2. Timing of Repair for CHD Requiring ECMO

Patients with CDH who require ECMO may undergo surgical repair at three different stages: before ECMO cannulation (pre-ECMO repair), during ECMO support (on-ECMO repair), or after ECMO decannulation (post-ECMO repair). Various authors have proposed specific timings for repair based on outcomes reported in their studies (Table 1).

### 2.1. Pre-ECMO Repair

Key et al. [15] advocated early repair before ECMO for left liver-up CDH, reporting a 95% survival rate in 21 patients who underwent repair within 60 h of life before ECMO, but only a 65% survival rate in 20 patients who started ECMO before repair. They developed an ECMO risk equation using pH, PCO_2_, and PO_2_ data in the first hour of life to evaluate cases retrospectively and suggested early repair before ECMO, particularly for severe cases with an ECMO risk exceeding 65%. This strategy is recommended only in experienced high-volume centers. Moscatelli et al. [16] presented a case of severe CDH successfully managed with veno-venous (VV) ECMO. They emphasized the importance of maintaining PDA patency with prostaglandin and lung recruitment, performing the repair before ECMO due to the cardiopulmonary deterioration caused by compression from herniated abdominal organs. They demonstrated successful left lung expansion and decreased pulmonary vascular resistance with lung recruitment during ECMO, suggesting that removing abdominal organs before ECMO initiation significantly contributed to the success of ECMO management.

### 2.2. On-ECMO Repair

Prabhu et al. [17] reported that six CDH cases repaired during ECMO maintained an activated clotting time (ACT) above 180 s. None of the cases had intraoperative bleeding, and five out of six were successfully weaned off ECMO and survived. The authors preferred early repair within 12–24 h during ECMO. Vaja et al. [18], analyzing data from the ELSO registry from 1989 to 2015, reported 82 (83.7%) repairs during ECMO, 13 (13.3%) before ECMO, and three (3%) after ECMO, with no statistically significant difference in survival among these groups. However, survival was higher in the pre-ECMO group (over 60%) compared to the cumulative (52.1%) and ECMO group (around 50%). Guner et al. [19] analyzed data from the ELSO registry from 2000 to 2015, developing models to predict mortality based on pre- and intra-ECMO factors in CDH patients. The timing of repair—pre-ECMO (Odds Ratio (OR) 1.06, 95% confidence interval (CI) 0.80–1.39) and on-ECMO (OR 0.87, 95% CI 0.72–1.04)—was not a significant factor affecting mortality. Despite the lack of statistical significance, the authors considered pre-ECMO repair a mortality factor, possibly due to the historical cognitive bias that surgery during the “honeymoon period” worsens PPHN, leading to ECMO. Glenn et al. [20] used data from the CDHSG registry (1995–2018) to examine early repair (within 72 h after cannulation) during ECMO versus no repair during ECMO (including post-ECMO repair and those never repaired). They found a statistically significant survival advantage in early repair during ECMO (87.1%) compared to no repair during ECMO (78.4%). However, ECMO duration was significantly longer in the early repair on-ECMO group (mean 270.2 h) than in the no-repair on-ECMO group (227.3 h). Long-term neurological and respiratory outcomes were not evaluated, leaving potential risks after prolonged ECMO runs unaddressed. Dao et al. [21] analyzed DHSG registry data (2000–2017) using propensity score matching and sensitivity analysis to study the impact of repair timing on CDH patients requiring ECMO. They classified centers by the frequency of repair during ECMO and the duration between the cannulation of ECMO and the repair. The “On-ECMO group”, defined by the highest quartile for ECMO repair frequency, had 88% of patients undergoing repair during ECMO (not 100%). The “After-ECMO group”, with the lowest quartile, still had 7.5% of patients undergoing repair during ECMO. Similarly, the “Late-ECMO group” (the highest quartile for duration between cannulation and repair) and the “Early-ECMO group” (lowest quartile) had median times to repair of 12.0 days and 2.0 days, respectively. They found a significantly lower mortality rate in the On-ECMO group compared to the After-ECMO group (OR 0.85, 95% CI 0.75–0.96) with PS matching analysis. The On-ECMO group significantly reduced the no-repair rate compared to the After-ECMO group (5.9% vs. 33.8%). However, with sensitivity analysis in which no repair was excluded, mortality was significantly higher in the On-ECMO group than in the After-ECMO group (OR 1.47, 95% CI 1.06–2.02). As for the Early- versus Late-ECMO group, they found that the Early-ECMO group had a significantly lower mortality rate than the Late-ECMO group with PS matching (OR 0.19, 95% CI 0.09–0.39); however, with sensitivity analysis, excluding non-repairs, there was no difference between those groups (OR 0.82, 95% CI 0.59–1.15). This may suggest that no repair would be a significant factor for mortality, and for patients requiring ECMO unrepaired, it might be beneficial to consider early repair; however, considering that repair during ECMO required a longer ECMO run (11 days vs. 9 days *p* = 0.001), the timing of repair should be individualized depending on the patient’s condition and the center’s experience. Despite these outcomes, this paper concluded that “*Although factors such as the patient’s clinical status and ECMO experience of individual treatment centers should be taken into account., centers should attempt to repair CDH early after ECMO cannulation as an effort to improve survival*”. Given the large dataset (1581 patients before exclusion) and publication in a high-impact journal, these findings may influence clinical practice. However, careful consideration of individual patient conditions and center experience is still essential when adopting this approach. Most of the recent reviews [10,11,26,27,28,29], as discussed later, recommended “early repair during ECMO” as the preferred approach. Nevertheless, implementing this strategy is far from straightforward, as it involves significant complexities that must be carefully considered.

### 2.3. Post-ECMO Repair

Partridge et al. [22] conducted a single-center analysis over nine years (2004–2012), showing that of 77 CDH patients requiring ECMO, 16 patients died without repair. Survival rates were 66% pre-ECMO, 44% during ECMO, and 100% post-ECMO. Significant bleeding complications were associated with on-ECMO repair. They concluded that delayed repair after ECMO might improve survival. Glenn et al. [23] used CDHSG data (1995–2016) to evaluate patients who had repairs after decannulation of ECMO and whether these patients returned to ECMO within 72 h after surgery. Among 2428 patients who experienced ECMO during this period, 859 (35.3%) were decannulated, and 668 (27.5%) underwent repair after decannulation, with only 0.9% (6/668) returning to ECMO within 72 h. They concluded that if patients could be stabilized on ECMO, repair should be performed after weaning from ECMO, avoiding complications such as hemorrhage with repair during ECMO. This report raised concerns that only 35.3% were decannulated, and 27.5% could be repaired. Most of those patients could never have had a chance for repair. The same authors later published their recommendation to focus on early repair during ECMO [18]. In the clinical setting, it would be difficult to determine whether a patient could wean from ECMO and have a chance to undergo repair or if they would never make it. Most patients would lose the opportunity to undergo repair, and ultimately, it would be difficult to save their lives. Delaplain et al. [24] used ELSO registry data (2000–2016) to perform propensity score matching to compare mortality and neurologic injury in 1112 cases between on-ECMO and post-ECMO repair. They found significantly higher mortality (OR 3.41, 95% CI 2.843–4.094) and neurological injury in on-ECMO compared to post-ECMO repairs. These findings suggest delaying repair until weaning from ECMO. However, as they described in the limitation, a significant number of patients (20%) could not be repaired, with nearly all (93%) dying. In practice, this strategy is difficult to apply, as it requires foreseeing whether a patient will successfully wean from ECMO during the ECMO run. Guner et al. [25] examined standardized mortality ratios (SMRs) across centers in the ELSO registry (2000–2019), identifying thirteen centers with significantly better SMRs and seven with worse SMRs. Better SMR centers performed more post-ECMO repairs (35%) and fewer no repairs (13%) compared to worse SMR centers (post-ECMO repair 14%, no repair 24.4%). They speculated that fewer no repairs and more post-ECMO repairs contributed to improved outcomes. They published data using ELSO (2000–2015) earlier to show no significant difference in mortality between pre- and post-ECMO repairs. However, they speculated that early repair during ECMO may change results in future studies.

Review articles [10,11,26,27,28,29] generally recommended on-ECMO repair with careful anticoagulant management to minimize the hemorrhage risks. Holden et al. [27] suggested that early repair offers physiological benefits. However, the timing definitions vary. Mchoney et al. [10] proposed early repair “within two weeks”, while Vandewalle et al. [28] defined it as “48–72 h” after ECMO initiation. Martino et al. [26] emphasized that the optimal timing of on-ECMO repair remains uncertain. The ELSO recommendation statement guidelines for the management of CDH requiring ECMO [29] are complex: for patients who can be decannulated or weaned off ECMO, repair after decannulation may be beneficial; for patients with severe CDH, early on-ECMO repair may be advantageous to avoid missing the chance for repair. In a clinical setting, deciding the timing of repair for those requiring ECMO can be immensely challenging.

## 3. Discussion

The following considerations are crucial for patients with CDH.

1. Repair should be delayed until the patient is hemodynamically stable.
2. If conventional management, including optimal ventilator settings, inotropic support, and pulmonary vasodilators, fails to stabilize the patient, ECMO becomes the next option as a last resort, improving oxygenation and acidosis while allowing the lungs to rest, potentially alleviating pulmonary hypertension. However, during ECMO, herniated organs may swell, increasing lung and heart compression, which could counteract the benefits of ECMO.
3. Reducing herniated organs into the abdomen can relieve lung and heart compression, improving systemic circulation and PPHN. However, the following must be considered:
  - Surgery before ECMO without stabilization risks complications such as cardiac and respiratory decompensation due to the invasiveness of surgery and thoracic configuration changes.
  - Surgery during ECMO risks severe, potentially fatal hemorrhage.
  - Surgery after ECMO may be safer, but risks losing the repair opportunity if the patients cannot stabilize, ultimately leading to death (Table 2).

### Proposal of Two-Staged Approach

A two-staged approach is proposed to address these challenges and has been successfully applied in a previously reported case by the author [14]. When ECMO is necessary, a laparotomy is first performed to reposition the herniated intrathoracic organs back into the abdominal cavity. The diaphragmatic defect is intentionally left open, and a wound retractor (e.g., Alexis^®^ Wound Retractor XS, Applied Medical, Rancho Santa Margarita, CA, USA) is used to create a silo, leaving a portion of the intestines outside the abdominal cavity. This reduces abdominal pressure while preventing re-herniation of the organs into the thoracic cavity. After a successful ECMO run and weaning from ECMO, the wound retractor is removed, and the diaphragmatic defect is closed with either a prosthetic patch or an abdominal muscle flap, ensuring a more stable surgical environment. In cases where an abdominal muscle flap is considered for repair, as the author reported, during the initial laparotomy, even though a left subcostal skin incision is made, the fascia is cut longitudinally along the lateral edge of the rectus abdominis to open the abdominal cavity. This preserves the transverse and internal oblique abdominal muscles for later repair.

This strategy might benefit severe CDH patients needing ECMO. However, some pediatric surgeons may be concerned about the potential for re-herniation, particularly in cases with large diaphragmatic defects. This concern might stem from their experiences with gastroschisis and large omphalocele, where the intestines naturally return to the abdominal cavity over time. In our clinical experience, although the intestines gradually returned to the abdominal cavity during ECMO, during the second surgery, we observed that organs such as the spleen, colon, and stomach, which are normally positioned subphrenically on the left side of the diaphragm, were elevated into the thoracic cavity due to the diaphragmatic defect. The majority of the intestines remained within the abdominal cavity. This suggests that without increased intra-abdominal pressure, the abdominal organs tend to remain in their anatomical positions within the abdominal cavity. Furthermore, the risk of re-herniation is minimized when the patient is fully sedated and positive airway pressure is carefully managed during ECMO. The use of a silo effectively reduces intra-abdominal pressure, maintaining organ stability. This is particularly important for large defects, where final closure with a prosthetic patch or muscle flap should be required post-ECMO.

In addition, the two-staged approach also offers a significant advantage in preventing abdominal compartment syndrome (ACS), which can occur after diaphragmatic defect repair. Creating a silo allows for the gradual adaptation of the intestines back into the abdominal cavity, reducing the risk of sudden increases in intra-abdominal pressure. This gradual reduction can help avoid the development of ACS and provides the abdominal cavity with sufficient time to expand and accommodate the intestines more naturally. This benefit is particularly crucial for preventing the need for extensive abdominal wall reconstruction following ECMO weaning.

However, potential disadvantages still include the risk of re-herniation even with a silo, as well as the possibility of cardiac decompensation and hemorrhage during ECMO. Given its potential disadvantages, this approach should be considered an ECMO standby option in high-volume medical centers. Nonetheless, the author believes a two-staged approach is worth further exploration to accumulate evidence for this challenging congenital disorder.

## 4. Conclusions

A two-stage approach, involving the reduction in herniated intrathoracic abdominal organs to relieve compression on the heart and lungs, followed by the creation of a silo before ECMO initiation and defect closure after ECMO decannulation, may offer a potential solution to this surgical dilemma.

## 5. Future Direction

- Determining the optimal timing of surgery continues to be a critical challenge among severe CDH patients requiring ECMO.
- While early repair during ECMO with careful anticoagulant management is currently recommended, the definition of “early” remains variable, and no significant improvements in mortality have been observed.
- Further accumulation of cases utilizing the two-staged approach—reducing herniated organs and creating a silo prior to ECMO initiation, followed by defect closure after ECMO weaning—could provide evidence of the approach’s efficacy and safety. Given the rarity of CDH cases requiring ECMO, a prospective multicenter cohort study would be ideal. Different centers could adopt specific strategies, such as early ECMO repair or the two-staged approach, allowing for a comparative analysis of outcomes. This research could help determine the optimal surgical timing and improve survival in these challenging cases.

## Figures and Tables

**Table 1:** Summary of surgical timing and outcomes for CDH requiring ECMO.

Study	Preferred/Recommended Approach	Key Findings
Key et al. [15]	Pre-ECMO	95% survival in 21 patients repaired pre-ECMO; 65% survival in 20 patients repaired during ECMO
Moscatelli et al. [16]	Pre-ECMO	Case successfully managed with VV-ECMO; repair before ECMO improved cardiopulmonary function
Prabhu et al. [17]	On-ECMO	6 cases; ACT > 180 s; 5 of 6 survived
Vaja et al. [18]	On-ECMO	ELSO data (1989–2015); 83.7% repaired during ECMO, 13.3% pre-ECMO, and 3% post-ECMO; no significant survival difference
Guner et al. [19]	On-ECMO	ELSO data (2000–2015); timing of repair not a significant mortality factor
Glenn et al. [20]	On-ECMO	CDHSG data (1995–2018); 87.1% survival with early ECMO repair vs. 78.4% with no repair; longer ECMO duration with early repair
Dao et al. [21]	On-ECMO	CDHSG data (2000–2017); early-ECMO group had lower mortality vs. late-ECMO group; no difference after, excluding non-repair
Partridge et al. [22]	Post-ECMO	77 cases; 44% survival with on-ECMO repair; 100% survival in post-ECMO group
Glenn et al. [23]	Post-ECMO	CDHSG data (1995–2016); 35.3% decannulated and 27.5% repaired post-decannulation; only 0.9% returned to ECMO within 72 h
Delaplain et al. [24]	Post-ECMO	ELSO data (2000–2016); 1112 patients; higher mortality and neurological injury in on-ECMO vs. post-ECMO repair
Guner et al. [25]	Post-ECMO	ELSO data (2000–2019); better SMR centers had more post-ECMO repairs (35%) and fewer no repairs (13%) than worse SMR centers

**Table 2:** Surgical dilemmas for CDH requiring ECMO.

Surgical Timing	Pros	Cons
Pre-ECMO	Relieves lung and heart compression	Risk of cardiac and respiratory decompensation due to unstable condition
On-ECMO	More stabilized cardiopulmonary condition	Risk of severe, potentially fatal hemorrhage due to anticoagulation
Post-ECMO	Stabilized condition	May lose the opportunity for repair
